# Aneurism of the Right Carotid and Subclavian Arteries; Ligation of the Arterial Innominata

**Published:** 1859-12

**Authors:** E. S. Cooper

**Affiliations:** San Francisco


### MISCELLANEOUS MEDICAL INTELLIGENCE.

### ANEURISM OF THE RIGHT CAROTID AND SUBCLAVIAN ARTERIES; LIGATION OF THE ARTERIAL INNOMINATA. BY E. S. COOPER, M.D., SAN FRANCISCO.

The October number of the *American Journal* contains a report of this case, if report it may be called, which omits nearly every important fact connected with the history of the case, the seat and extent of the disease, its effects, etc.

Dr. Cooper states, "I long tried to make room for the application of a ligature to the Arteria Innominata without removing more important structures." (?) "Finding this, however, almost, or quite impossible, I proceeded to remove the summit of the sternum and the sternal extremity of the clavicle." "The patient gradually sank until the 9th, when he expired." Among the *post mortem* appearances was observed a tubercularization of the right lung.

We notice this operation, to say that it is one which cannot receive the approbation of any judicious surgeon.

Ligature of the arteria innominata has been performed nine times. In all the result was fatal, viz:

1. Dr. Mott, May 11, 1818.—For aneurism of the subclavian and carotid arteries, extending to the innominata. Death the twenty-fifth day, ferny coming from the distal side of the ligature.

2. Graeffe of Berlin, for aneurism of the subclavian artery. Death the sixty-seventh day from hemorrhage.

3. Bland of Sidney, New South Wales. Death the eighteenth day, from hemorrhage from the subclavian artery.

4. Richard Wilmot Hall, for aneurism of the subclavian artery. Death in five days, from hemorrhage.

5. Lizars, for aneurism of the subclavian artery. Death the twenty-first day.

6. Kuhl of St. Petersburg. Death the third day.

7. Arena, surgeon of the Emperor of Russia. Death five days after the operation.

8 and 9. Bugalsky. Details not given. Death soon after the operations.

No notice of the situation of the aneurism, or the state of the artery, are given in the autopsy.

Cases of this kind, published without comment, and thus partly endorsed by journalists, have given rise to the term "audace Americaine," used by Trousseau. If editors, in giving currency to this and similar reports, would express their opinions of the propriety of such operations, it is likely that fewer would be done, and the responsibility be thrown upon the individuals who, without any prospect of benefit to their patients, think fit to resort to them.

